# Prevalence of Potential Drug–Drug Interaction Risk among Chronic Kidney Disease Patients in a Spanish Hospital

**DOI:** 10.3390/pharmaceutics12080713

**Published:** 2020-07-30

**Authors:** Gracia Santos-Díaz, Ana María Pérez-Pico, Miguel Ángel Suárez-Santisteban, Vanesa García-Bernalt, Raquel Mayordomo, Pedro Dorado

**Affiliations:** 1 Biosanitary Research Institute of Extremadura (INUBE), University of Extremadura, 06006 Badajoz, Spain; grsantosd@unex.es (G.S.-D.); miguelangel.suarez@salud-juntaex.es (M.Á.S.-S.); 2 Department of Nursing, University of Extremadura, 10600 Plasencia, Spain; aperpic@unex.es; 3 Nephrology Department, Virgen del Puerto Hospital, Servicio Extremeño de Salud, 10600 Plasencia, Spain; vanesa.garcia@salud-juntaex.es; 4 Department of Anatomy, Cellular Biology and Zoology, University of Extremadura, 10600 Plasencia, Spain; rmayordo@unex.es

**Keywords:** chronic kidney disease, drug–drug interactions, polypharmacy, adverse drug reactions, Lexicomp

## Abstract

Chronic kidney disease (CKD) is a major health problem worldwide and, in Spain, it is present in 15.1% of individuals. CKD is frequently associated with some comorbidities and patients need to be prescribed multiple medications. Polypharmacy increases the risk of adverse drug reactions (ADRs). There are no published studies evaluating the prevalence of potential drug–drug interactions (pDDIs) among CKD patients in any European country. This study was aimed to determine the prevalence, pattern, and factors associated with pDDIs among CKD patients using a drug interactions program. An observational cross-sectional study was carried out at Plasencia Hospital, located in Spain. Data were collected among patients with CKD diagnoses and pDDIs were assessed by the Lexicomp^®^ Drug Interactions platform. Data were obtained from 112 CKD patients. A total number of 957 prescribed medications were acknowledged, and 928 pDDIs were identified in 91% of patients. Age and concomitant drugs were significantly associated with the number of pDDIs (*p* < 0.05). According to the results, the use of programs for the determination of pDDIs (such as Lexicomp^®^) is recommended in the clinical practice of CKD patients in order to avoid serious adverse effects, as is paying attention to contraindicated drug combinations.

## 1. Introduction

As specified by the Kidney Disease Improving Global Outcomes (NKF KDIGO) guidelines [1], chronic kidney disease (CKD) is defined as abnormalities of kidney structure or function, present for more than three months, with implications for health. CKD is a general term for various heterogeneous disorders affecting kidney structure and function with variable clinical presentations; in part, related to the cause, severity, and rate of progression. The glomerular filtration rate (GFR) is generally accepted as the best overall index of kidney function, and is classified into different stages (G1, G2, G3a, G3b, G4, and G5). The diagnostic criteria of CKD are those denominated as kidney damage markers or a threshold of GFR < 60 mL/min/1.73 m^2^ (GFR categories G3a–G5), or both, for more than three months.

CKD is a major health problem worldwide; in 2017, 1.2 million people died from CKD. Furthermore, between 1990 and 2017, the global all-age mortality rate from CKD increased by 41.5% [2]. In Spain, CKD is present in 15.1% of individuals and this prevalence is more than three times higher in men than in women (23.1% vs. 7.3%) and increases with age [3]. Diabetes and hypertension are the main causes of CKD in all high-income and middle-income countries and in many low-income countries [4]. Among other reasons, the prevalence of CKD is increasing worldwide due to the fact that the prevalence of both hypertension and diabetes is also rising. Diabetes is expected to increase by 69% in high-income countries and 20% in low-income and middle-income countries from 2010 to 2030 [5]. Regarding hypertension, it is predicted to increase by 60% from 2000 to 2025 [6]. Additionally, CKD is also associated with other comorbidities such as dyslipidemia, hyperuricemia, or cardiovascular disease [7], and patients need to be prescribed multiple medications.

Polypharmacy is usually defined as the concomitant prescription of five or more medications [8] and it is a major risk factor of drug–drug interactions, which increases with the number of prescribed drugs leading to 100% with eight or more medications [9]. The elderly are at risk for polypharmacy, and this fact increases the risk of adverse drug reactions (ADRs) from 13 to 58% with two and five medications, respectively. Seven or more medications increase the risk of ADRs to 82% [9].

Previous studies have evaluated the prevalence and severity of potential drug–drug interactions (pDDIs) using different drug–drug interaction programs among CKD patients from Brazil [10,11], India [12,13], Pakistan [14], Palestine [15], and Nigeria [16,17,18,19]; however, there are no published studies evaluating the prevalence of pDDIs among CKD patients in any European country.

Lexicomp^®^ (Wolters Kluwer Clinical Drug Information) is considered one of the best performing drug–drug interaction programs and it was reported to be highly sensitive and specific (around 90–100%). It focuses on the depth and duplication of information and it is a resource of choices for locating the mechanism of a drug–drug interaction [20,21,22]. 

This study was aimed to determine the prevalence, pattern, and factors associated with potential drug–drug interaction among CKD patients attending a hospital nephrology department using the drug interaction program Lexicomp^®^.

## 2. Materials and Methods 

### 2.1. Subjects

An observational cross-sectional study was carried out at Virgen del Puerto Hospital in Plasencia (Cáceres, Spain). All participants were patients attended by the nephrology department, and were invited to participate in the study. The inclusion criteria were: patients with CKD diagnosis, over the age of 18, and having signed an informed consent form. Data were collected during 2019 and included: age, gender, list of medications at the time of last clinic visit, comorbidities, and serum creatinine.

The study was performed in accordance with the principles of the Declaration of Helsinki of 1975, revised in 2013, and approved by the Clinical Research Ethics Committee, Cáceres (reference: MASR/2016), and the Bioethics and Biosecurity Committee, University of Extremadura (reference: 64/2016). 

The serum creatinine value was used to calculate eGFR (estimated glomerular filtration rate in mL/min/1.73 m^2^) and patients were classified following the criteria of the KDIGO Guideline 1 into different CKD stages: G1, eGFR ≥ 90; G2, eGFR 60–89; G3a, eGFR 45–59; G3b, eGFR 30–44; G4, eGFR 15–29; G5, eGFR < 15.

### 2.2. Methods

The electronic drug–drug interactions (DDIs) checking platform Lexicomp^®^ was used to evaluate patient medication regimens for pDDIs. The Lexicomp^®^ (Wolters Kluwer Health Inc. Riverwoods, IL, USA) database system provides accurate information about the risk, type, mechanism, and pattern of distribution of pDDIs. It also gives recommendations on how to prevent and manage DDIs if they occur. This software identifies and classifies pDDIs into five types according to the degree of clinical significance. Type A: no known interaction, Type B: minor or mild interaction, Type C: moderate or significant interaction, Type D: major or serious interaction, and Type X: contraindication or avoid combination.

### 2.3. Statistical Analysis

Descriptive statistics were used, and results were presented as percentages and frequencies. ANOVA Kruskal–Wallis test or Mann–Whitney t-test analyses were performed to evaluate the effect of covariates on the incidence of pDDIs. A *p*-value of less than 0.05 was considered statistically significant. All statistical analyses were performed using IBM SPSS v.22 (SPSS Inc., Chicago, IL, USA).

## 3. Results

### 3.1. Clinical and Demographic and Characteristics of Patients

Data were obtained from 112 CKD patients, 69 (61.6%) females and 43 (38.4%) males. The mean age of this study population was 77.1 ± 10.4 years: 11 patients (10.0%) were between 30 and 60 years, 8 (7.1%) were between 61 and 70 years, 44 (39.3%) were between 71 and 80 years, and 49 (43.7%) were older than 80 years (Table 1). 

The most common comorbid conditions (Table 1) were hypertension in 52 patients (46.4%), diabetes mellitus in 25 (22.3%), dyslipidemia in 33 (29.5%), anemia in 13 (11.6%), and hyperuricemia in 11 (9.8%).

### 3.2. Prevalence and Pattern of Potential Drug–Drug Interactions

A total number of 957 prescribed medications were identified. The minimum number of prescribed medications per patient was 1, the maximum was 17, and the mean number was 8.6 ± 3.4 medications. Only one patient was not taking any medication. The most commonly prescribed medications were omeprazole (30.6%), acetaminophen (30.6%), salicylic acid (26.1%), bisoprolol (25.2%), furosemide (22.5%), and allopurinol (21.6%).

Among 111 individuals 928 pDDIs were identified, and 67 (60.3%) patients showed 1–10 pDDIs, while 34 (30.6%) presented more than 10. Only 10 patients (9%) did not have any interaction (Table 2).

According to the Lexicomp^®^ severity classification, 11 (1.2%) pDDIs were Type A (no known interaction), 84 (9.1%) were Type B (mild severity), 717 (77.3%) were Type C (moderate severity), 106 (11.4%) were Type D (major severity), and 10 (1.1%) were Type X (avoid drug combination) (Table 3).

Table 4 shows the most frequent pDDIs by severity group: levothyroxine + omeprazole with 9 cases in the Type B group (10.7%), acenocoumarol + omeprazole with 11 cases in Type C (1.5%), and acenocoumarol + allopurinol with 8 cases in Type D (7.5%).

In addition, Type X (avoid drug combination) pDDIs were found in 10 CKD patients (Table 5).

**Figure 1:**
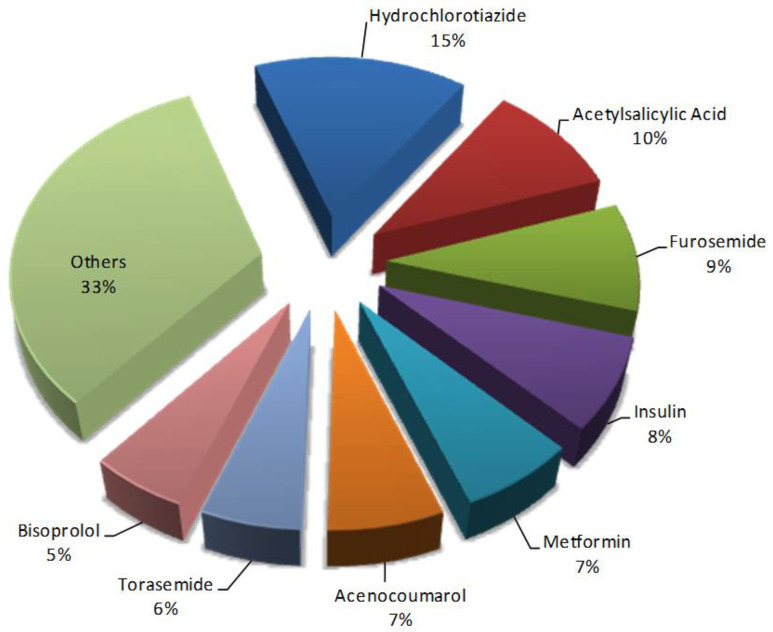
Frequency of main drugs with potential drug–drug interactions (n = 928).

It was also observed that some drugs were present in a large number of pDDIs such as hydrochlorothiazide (15%), acetylsalicylic acid (10%), or furosemide (9%). The most frequent drugs present in pDDIs in the study group are shown in Figure 1.

### 3.3. Factors Associated with Potential Drug–Drug Interactions on CKD Patients

Age and concomitant drugs were significantly associated with the number of pDDIs (*p* < 0.05; Table 6). In contrast, demographic and clinical variables, such as gender, CKD stage, or the number of chronic comorbid diseases were not significantly associated with the number of pDDIs (Table 6).

## 4. Discussion 

### 4.1. Frequency and Severity of Potential Drug–Drug Interactions

Table 7 shows previous reports in which the prevalence and severity of pDDIs has been evaluated on CKD patients. It is remarkable that there are two studies published in 2017 that were carried out in different hospitals in Nigeria, with different numbers of individuals, but their results are practically identical despite using different analysis tools [17,18].

As mentioned, most of the pDDIs were Type C (moderate severity), and 12.5% were Type D (major severity) and Type X (avoid drug combination). These data are similar to those observed in a previous study in Palestinian patients [15]. Other studies highlighted differences in the frequency of pDDIs types (Table 7). Among other causes, the variability in the reported pDDIs could also be a consequence of using different screening platforms to analyze potential drug interactions. In our case, we used Lexicomp^®^, which classifies pDDIs in different levels of severity. However, other software (Micromedex Drug-Reax, Medscape Drug interaction checker, etc.) for similar drug combinations perform dissimilar categorizations, or find different pDDIs. 

Even though the majority of pDDIs reported in this study were Type C, it is necessary to closely monitor patients in order to identify adverse events. Moreover, major severity drug interactions and avoided drug combinations present a high risk to the health of patients and, consequently, physicians or clinical pharmacists must analyze, detect, and early prevent pDDIs.

In the present study, the majority of the patients were in CKD stage 3, and only 4.5% of the total were in CKD stage 5 or hemodialysis. This result is comparable with another study from Brazil [10] in which patients in CKD stage 5 represented 6.6% of the total sample. However, in the remaining previous studies, most of the patients were in stage 5 or hemodialysis (Table 7). This could affect the number and type of prescribed treatment and, therefore, the pDDI. The present study did not only focus on patients on hemodialysis, but on all patients with CKD.

### 4.2. Factors Associated with Potential Drug–Drug Interactions

Regarding comorbid conditions, these appeared in 91 patients (81.2%), and the most frequent were hypertension (44.6%), dyslipidemia/hypercholesterolemia (28.6%), and diabetes mellitus (22.3%).

The prevalence of hypertension and diabetes in previous studies [10,13,14,15] were higher than those found in the present study. In addition, each country could have implemented different clinical guidelines for similar disease conditions, which results in the prescription of different drugs and thereby other pDDIs. The selected hospital is a reference hospital with a nephrology unit similar and representative of most hospitals in the country. The percentage of patients with chronic kidney disease is also similar to those other nephrology units in Spain.

In the present study, the mean age of patients was higher (77.1 ± 10.4 years) than in the rest of the studies, which reported mean age data from 38.3 ± 16.8 to 59.1 ± 14.7 years (Table 7). Polypharmacy prevalence increases with advancing age [23], and hence also pDDIs. Furthermore, people aged 80 and over are still much more likely to have DDIs [24]. Therefore, the main reasons for the differences found in the present study, comparing to most of the previous studies, could be the different CKD stages, age, or the country of patients. These factors could affect the number and type of prescribed drug treatment and, therefore, the number and severity of pDDIs. On the other hand, the use of different software to evaluate the pDDIs in the reported studies (Table 7) could lead to differences of the severity of pDDIs. Furthermore, many of the analyzed drugs that appear in Lexicomp^®^ do not appear in some other databases. 

This could be one of the reasons for variability in the pattern of frequency and severity of pDDIs observed in the present study compared to previous studies.

## 5. Conclusions

The frequency and severity of pDDIs could be affected by the type and number of drugs per patient, which, at the same time, could be influenced by comorbidities and age. On the other hand, the advancement of CKD increases the risk of a major cardiac event and the possibility of hospitalization, which increases the number of medications [25,26].

It should be noted that in CKD patients, the association of medications is sometimes inevitable, and according to the present results, the use of programs for determination of pDDIs (such as Lexicomp^®^) are recommended in clinical practice for CKD patients in order to avoid serious adverse effects, paying attention to the contraindicated drug combinations. Therefore, as a way to classify and identify pDDIs according to interaction risk, severity, and reliability, it would be convenient to consider and evaluate pDDIs in clinical practice in order to avoid or prevent some avoidable adverse effects.

## Figures and Tables

**Table 1:** Characteristics of the study population (n = 112).

Characteristics	N (%) or Mean ± SD
Female	69 (61.6)
Male	43 (38.4)
Mean age (years)	77.1 ± 10.4
Age group (years)	
30–60	11 (10.0)
61–70	8 (7.1)
71–80	44 (39.3)
>80	49 (43.7)
CKD stage	
1	10 (8.9)
2	15 (13.4)
3a	34 (30.3)
3b	33 (29.5)
4	15 (13.4)
5	5 (4.5)
Comorbidities	91 (81.2)
Hypertension	52 (46.4)
Diabetes mellitus	25 (22.3)
Dyslipidemia/hypercholesterolemia	33 (29.5)
Anemia	13 (11.6)
Hyperuricemia	11 (9.8)
Mean prescribed drugs per patient	8.6 ± 3.4
Number of prescribed drugs	
≤5	22 (19.6)
6–10	59 (53.2)
11–15	29 (25.9)
≥16	2 (1.8)

**Table 2:** Frequency of potential drug–drug interactions (pDDIs) per patient (n = 111 *).

Number of pDDIs	N	%
None	10	9.0
1–5	40	36.0
6–10	27	24.3
11–15	15	13.5
16–20	11	9.9
21–25	4	3.6
>25	4	3.6

* One patient had not taken any drugs.

**Table 3:** Severity of potential drug–drug interactions (pDDIs; n = 928) among studied chronic kidney disease (CKD) patients.

Severity of pDDIs	N	%
Type A (No known interaction)	11	1.2
Type B (mild severity)	84	9.1
Type C (moderate severity)	717	77.3
Type D (major severity)	106	11.4
Type X (avoid drug combination)	10	1.1

**Table 4:** Most frequent potential drug–drug interactions (pDDIs) by severity group.

Severity of pDDI	N	PDDIs	Frequency (%)
Type B (mild severity)	84	Levothyroxine + Omeprazole	10.7
		Levothyroxine + Furosemide	9.5
		Acenocoumarol + Spironolactone	7.1
Type C (moderate severity)	717	Acenocoumarol + Omeprazole	1.5
		Ferrous Sulfate + Omeprazole	1.3
		Metformin + Acetylsalicylic Acid	1.3
Type D (major severity)	106	Acenocoumarol + Allopurinol	7.5
		Levothyroxine + Ferrous Sulfate	4.7
		Tramadol + Trazodone	3.8

**Table 5:** Potential drug–drug interactions Type X (avoid drug combination) found in the studied CKD patients.

Drug–Drug Interaction	Potential Adverse Effects	Severity	Reliability Rating
Amitriptyline + Aclidinium	Aclidinium may enhance the anticholinergic effect of Anticholinergic Agents	Major	Fair
Doxazosin + Dutasteride and Tamsulosin	Alpha1-Blockers may enhance the antihypertensive effect of other Alpha1-Blockers	Major	Fair
Dutasteride And Tamsulosin + Tamsulosin	Alpha1-Blockers may enhance the antihypertensive effect of other Alpha1-Blockers	Major	Fair
Levosulpiride + Solifenacin	Anticholinergic Agents may diminish the therapeutic effect of Levosulpiride	Major	Fair
Aclidinium and Formoterol + Propranolol	Beta-Blockers (Nonselective) may diminish the bronchodilatory effect of Beta2-Agonists	Major	Fair
Metamizole + Dexketoprofen	Dexketoprofen may enhance the adverse/toxic effect of Nonsteroidal Anti-Inflammatory Agents	Major	Fair
Tramadol and Acetaminophen + Buprenorphine	Opioids (Mixed Agonist/Antagonist) may diminish the analgesic effect of Opioid Agonists	Major	Good
Atenolol + Rivastigmine	Rivastigmine may enhance the bradycardic effect of Beta-Blockers	Moderate	Fair
Bisoprolol + Rivastigmine	Rivastigmine may enhance the bradycardic effect of Beta-Blockers	Moderate	Fair
Dexketoprofen + Acetylsalicylic Acid	Salicylates may enhance the adverse/toxic effect of Dexketoprofen. Dexketoprofen may diminish the therapeutic effect of Salicylates. Salicylates may decrease the serum concentration of Dexketoprofen	Major	Fair

**Table 6:** Potential drug–drug interactions (pDDIs) among 111 * CKD patients according to demographic and clinical variables groups.

Variable	Number	Median (25%–75% Percentile)	*p*–Value ^1^
**Gender**			0.5768 ^2^
Female	68	7 (2–12.7)	
Male	43	7 (2–11)	
**Age**			0.0191
30–60	11	2 (0–5)	
61–70	8	3.5 (0.5–7.7)	
71–80	44	8 (3.2–16)	
>80	48	8 (3–11)	
CKD stage **			0.4930
G1	10	3.5 (0.7–12.7)	
G2	15	8 (3–12)	
G3a	34	5 (1–9.2)	
G3b	33	8 (3–13)	
G4	14	4 (3–13)	
G5	5	11 (4–17.5)	
**Concomitant drugs**			<0.0001
≤5	21	1 (0–2)	
6–10	59	6 (4–9)	
11–15	29	16 (11–20)	
>15	2	26 (16–36)	
**Chronic comorbid disease**			0.0611
0	20	6.5 (3.2–11)	
1	21	7 (2–12.5)	
2	20	6.5 (3–10.7)	
3	10	12 (7.2–20.5)	
4	17	7 (1–17.5)	
≥5	23	4 (1–7)	

* One patient had not taken any drugs; ** according to the classification of chronic kidney disease from Kidney Disease: Improving Global Outcomes (KDIGO); ^1^ ANOVA Kruskal–Wallis test; ^2^ Mann–Whitney *t*-test.

**Table 7:** Previous studies in which prevalence and severity of potential drug–drug interactions has been evaluated on CKD patients.

Study	Number of Patients (% Female)	Years (Mean ± SD)	Country	CKD Patients on Stage 5 or Hemodialysis (%)	Software for Potential DDI	Number of Drugs per Patient (Mean ± SD)	Most Frequent Drug Combinations with Potential DDIs (%)	Number of Patients with Potential DDIs Contraindicated (%)
Rama et al. [12].	205 (25.8%)	48.6 ± 16.2	India	68.5%	Micromedex	12.1 ± 6.3	Ascorbid Acid + Cyanocobalamine (12.4%) Clonidine + Metoprolol (3.8%) Amlodipine + Metoprolol (3.4%) Insulin + Metoprolol (2.9%)	0 (0.0%)
Marquito et al. [10].	558 (45.3%)	n.s.	Brazil	6.6%	Micromedex	5.6 ± 3.2	Furosemide + Aspirin (7.8%) Enalapril + Furosemide (5.9%) Captopril + Furosemide (5.1%) Enalapril + Losartan (3.7%)	5 (0.9%)
Sgnaolin et al. [11].	65 (50.8%)	59.1±14.7	Brazil	100%	Micromedex	6.3 ± 3.1	Calcium Carbonate + Atenolol (8.0%) Calcium Carbonate + Ferrous Sulfate (8.0%) Calcium Carbonate + Ticlopidine (6.3%) Enalapril + Eritropoietin (4.5%)	2 (3.1%)
Hegde et al. [13].	120 (45%)	58.5 ± 8.4	India	n.s.	Medscape Drug interaction checker	9.4 ± 3.9	Sodium Bicarbonate + Ferrous Sulfate (8.9%) Calcium Carbonate + Ferrous Sulfate (5.5%) Aspirin + Carvedilol (5.5%) Sodium Bicarbonate + Allopurinol (5.5%)	0 (0.0%)
Al-Ramahi et al. [15].	275 (45.1%)	50.7 ± 15.9	Palestina	100%	LexiComp	7.9 ± 2.4	Calcium Carbonate + Amlodipine (12.3%) Calcium Carbonate + Aspirin (8.2%) Aspirin + Furosemide (7.9%) Aspirin + Enoxaparin (4.3%)	2 (0.7%)
Olumuyiwa et al. [18].	123 (33.3%)	53.8 ± 16.0	Nigeria	69.9%	Lexi-Interact database	10.1 ± 4.0	Calcium Carbonate + Ferrous Sulfate (8.4%) Folic Acid + Furosemide (3.4%) Calcium Carbonate + Calcidol (3.2%) Vitamin E + Ferrous Sulfate (3.0%)	1 (0.8%)
Fasipe et al. [17].	123 (48.8%)	53.8 ± 16.0	Nigeria	69.9%	Medscape Drug interaction checker	10.3 ± 3.9	Calcium Carbonate + Ferrous Sulfate (9.9%) Folic Acid + Furosemide (3.4%) Calcium Carbonate + Calcidol (3.2%) Vitamin E + Ferrous Sulfate (3.0%)	1 (0.8%)
Saleem et al. [14].	209 (39.2%)	38.3 ± 16.8	Pakistan	74.2%	Micromedex Drug-Reax	n.s.	Ferrous Sulfate + Omeprazole (5.8%) Calcium/Vitamin D + Ciprofloxacin (4.8%) Captopril + Furosemide (4.1%) Calcium Gluconate + Ceftriaxone (3.6%)	28 (13.4)
Adibe et al. [16].	169 (52.1%)	51.0 ± 14.9	Nigeria	28.4%	Medscape Drug interaction checker	6.1 ± 2.0	Lisinopril + Furosemide (9.1%) Furosemide + Calcium Carbonate (7.2%) Calcium Carbonate + Lisinipril (6.1%) Aspirin + Furosemide (4.6%)	0 (0.0%)
Okoro and Farate [19].	201 (66%)	49.5 ± 14.5	Nigeria	69.2%	Omnio drug interaction checker	5.8 ± 1.5	Calcium Carbonate + Ferrous Sulfate (45.8%) Lisinopril + Furosemide (7.7%) Captopril + Furosemide (6.6%) Captopril + Spironolactone (6.6%)	5 (2.5%)
**Present study**	111 (61.3%)	77.1 ± 10.4	Spain	4.5%	LexiComp	8.6 ± 3.4	Acenocoumarol + Omeprazole (1.1%) Ferrous Sulfate + Omeprazole (1.0%) Metformin + Aspirin (1.0%) Levothyroxine + Omeprazole (1.0%)	10 (9.0%)

n.s.: not studied.
